# Annual Address of the President of the Illinois State Medical Society, Read at the Annual Meeting, in Vandalia, June 4th, 1856

**Published:** 1856-11

**Authors:** 


### EDITORIAL DEPARTMENT.

---

*Annual Address of the President of the Illinois State Medical Society, read at the Annual Meeting, in Vandalia, June 4th,* 1856. By N. S. DAVIS, M. D.

We beg of our readers to turn back to our eclectic department, and give a careful perusal to the argument of Prof. DAVIS, on the treatment of consumption, published under the head given above.

The editorial article in our April number, which forms the text of this address, was a hasty production, stating our personal convictions without argument to sustain them. We distinctly stated in the brief page devoted to that article, that we wished only to state our belief, not to brace it up by argument. Unfortunately, that page, written under an urgent sense of neglected duty in not having given sufficient prominence to the new idea in the treatment of consumption, has, as stated by Prof. Davis, been very largely copied, and with all its imperfections, its utter lack of argument, and its warm assertion of personal belief, has gone to the bar of professional opinion. One after another of our leading journals have copied it, with brief but emphatic endorsement, until it has thus assumed an importance not originally its own, and has at last called out the able and dignified reply of Prof. Davis. It has thus gone before a learned and highly respectable State society, and the time has come when we must defend our position. Fortunately, it was originally taken with due consideration, and is woith the defence.

In the first place, we adopt the conclusions of Prof. Davis as to the action of alcohol, although we might be inclined to dispute the third. The important proposition is that alcohol "diminishes the excretion of carbonic acid from the lungs, diminishes the change of color from venous to arterial blood; and diminishes generally the organic changes in the system."

And these, let us say, are the reasons, in part, why we prescribe it. Let it be recollected that in consumption we have a rapid pulse, a high temperature subject to sudden chill and congestion; that the excretion of carbon from the lungs is morbidly active; that the blood has, even in the veins, a vivid arterial color, and is highly oxygenated, and that the "organic changes in the system," the processes of waste particularly, are morbidly active, and we have good and sufficient reason for the administration of alcohol.

As to Dr. Davis' history of cases of consumptive drunkards, we could readily add to the number. We are not so strong a temperance man that we can draw no line between use and abuse; we have never advocated drunkenness as a prophylactic against any disease: albeit we have sometimes prescribed alcohol for delirium-tremens. The drunkard may have tubercle —ought to have it. He neglects, in his absurd appetite, all those rules of diet, those regular habits, which are essential to health. He destroys his digestive functions by over-stimulation, and in their loss of tone, in the creation of dyspepsia, he lays the foundation for tubercle.

Dr. Davis himself rejects the old-time theories of consumption; he does not believe in changes of temperature, nor in the congestive theory, and he virtually admits that in the recuperation of the system generally, in the supply of fatty constituents, in the building up of the digestive and assimilative functions, is to be found the true cure, if any there be, for tuberculosis. The only point of difference between us, then, is that he would strike out of the list of remedies all alcoholic stimulants, and would retain the fat food, the exercise, the free air, and the tonics.

In order to make this point the stronger, he has been unconsciously betrayed into a misstatement of our position. He quotes the case of our friend, Dr. G., cured by whisky punch, though he doubts the permanence of that cure. Two years have elapsed, and we have the pleasure of knowing that Dr. G. is not so far overcome, either by tuberculosis or punch, but that he is able to perform an immense amount of labor. But he does not quote the case of our other friend, Dr. B., who cured his hæmoptysis by crossing the Atlantic in April, in storm and hardship, and in care of 600 emigrants. To-day Dr. B. is engaged in the dissection-room and is in good health. We gave this case, in which no alcohol was used, as well as that of Dr. G., in which much alcohol was used, and the inference therefrom was, that we were not advocates of the alcoholic treatment alone.

We did say that it was far better to take cold than to get dyspepsia, and that brings us to the true argument.

It is our belief that consumption occurs, in those predisposed to it, by that form of dyspepsia which prevents the proper digestion of fatty food. Now it is known that the pancreas is the gland which governs this part of the digestive process, that a healthy pancreatic fluid ensures the digestion of fat, and *vice-versa.* Suppose, then, that the secretions of the stomach are so acid as to more than neutralize the alkalinity of the ingesta and of the bile, and leave a surplus, which neutralizes also the alkalinity of the pancreatic fluid. When this happens, as it not unfrequently does, the digestion of fat is impossible; not that the alkalinity of the pancreatic fluid is the sole condition of its solvent power, but that it is *one* of the normal conditions, and its absence must therefore impair the functions.

In such a case as this, the presence of a little alcohol in the stomach will subserve one or all of three purposes.

*First.* It is, according to Liebeg and the most competent chemists of the age, a carbonaceous food.

*Second.* It may give a fillip to the powers of the pancreas, enabling it for the time to secrete properly.

*Third.* The alcohol itself may act as a substitute for the pancreatic juice, transferring the digestion of fat from the duodenum to the stomach, and emulsionizing the fat, so that it shall be capable of absorption.

On either one of these probabilities we are willing to prescribe alcohol.

Anothor reason is found in the kindly effect of a moderate amount of alcohol on the entire digestive process, its control over the stomach by the stimulus it affords to the cœliac plexus.

These, then, are our arguments in favor of the administration of alcoholic or fermented liquors in consumption. Dr. Davis' objections may now come in and receive their full weight.

The first and most serious objection is that urged by the St. Louis Journal, which at the same time endorses the curative powers of the treatment. *The habit of drinking may be engendered.* Do we think of this in typhoid fever? Does it prevent our administration of opium? Have we a right to withhold an important curative means, lest the patient may abuse it to his own permanent injury? Have we a right to assume that our patient has no control over his appetites? And dare we preserve his morals at the expense of his life? Had Cain been innocent of the murder of Abel, his answer "Am I my brother's keeper?" would have been appropriate and excusable. This is a question for the judgment of the individual practitioner in the individual case, and the writer or teacher has no right to do more than to convey a word of caution.

The other objections offered by Dr. Davis, with the exception of those drawn from the pathology of the disease, and which we have already sufficiently answered, are founded upon the abuse of the remedy, or upon its exclusive use and isolation from the more important features of the treatment. We advocate, not the alcoholic treatment of tuberculosis, but the fat treatment. The latter is the essential, the former the adjuvant. We can do without the alcohol, sometimes; without the fat, never.

We regard, then, alcohol as a most important auxiliary in the tonic treatment; but we look upon all that may in any way momentarily or permanently give vigor to the digestive and assimilative functions as remedies.

We are pleased that Prof. Davis has so highly complimented us by bestowing upon us, in the time and occasion of his address, an importance to which we lay no claim. We are further gratified that in his method of handling the question, he has preserved that dignity of manner and sincerity of purpose which are his birthright, and more than for either of these do we rejoice, that we differ, not in the curative idea, not in the general outline of treatment, but only in the use and recommendation of one of the auxiliary remedies.

